# What makes TMB an ambivalent biomarker for immunotherapy? A subtle mismatch between the sample-based design of variant callers and real clinical cohort

**DOI:** 10.3389/fimmu.2023.1151224

**Published:** 2023-05-25

**Authors:** Yuqian Liu, Shenjie Wang, Yixuan Wang, Yifei Li, Xiaoyan Zhu, Xin Lai, Xuanping Zhang, Xuqi Li, Xiao Xiao, Jiayin Wang

**Affiliations:** ^1^ School of Computer Science and Technology, Faculty of Electronics and Information Engineering, Xi’an Jiaotong University, Xi’an, Shaanxi, China; ^2^ Shaanxi Engineering Research Center of Medical and Health Big Data, Xi’an Jiaotong University, Xi’an, Shaanxi, China; ^3^ Department of Biomedical Engineering, Nanjing University of Aeronautics and Astronautics, Nanjing, China; ^4^ Department of General Surgery, The First Affiliated Hospital of Xi’an Jiaotong University, Xi’an, Shaanxi, China; ^5^ Geneplus Shenzhen, Shenzhen, China

**Keywords:** clinical immunology, tumor mutation burden, categorization thresholds, sequencing data analysis, bioinformatics tool, measurement error

## Abstract

Tumor mutation burden (TMB) is a widely recognized biomarker for predicting the efficacy of immunotherapy. However, its use still remains highly controversial. In this study, we examine the underlying causes of this controversy based on clinical needs. By tracing the source of the TMB errors and analyzing the design philosophy behind variant callers, we identify the conflict between the incompleteness of biostatistics rules and the variety of clinical samples as the critical issue that renders TMB an ambivalent biomarker. A series of experiments were conducted to illustrate the challenges of mutation detection in clinical practice. Additionally, we also discuss potential strategies for overcoming these conflict issues to enable the application of TMB in guiding decision-making in real clinical settings.

## 1 Introduction

Immunotherapy has altered cancer treatment paradigms as a result of substantial advancements in immune checkpoint blocking (1–3). Increasing numbers of advanced cancer patients benefit from immune-checkpoint inhibitor (ICI) therapy (4). Tumor mutation burden (TMB) has been intensively studied as the promising immunotherapy biomarker for patient selection (5, 6). TMB refers to the number of somatic mutations per megabase (7). Clinical studies have noted that patients with high TMB tend to benefit more from immunotherapy (8). The association of high TMB with improved patient responses and survival benefits after immunotherapy has been observed in urothelial cancer (9), small cell lung cancer (10), non-small-cell lung cancer (11), among others. The US FDA has also prioritized TMB as the recommended test for cancer patients (12).

In clinical practice, it is only practical for physicians when TMB levels can effectively categorize patients into different risk groups with varying therapeutic benefits. However, here, TMB remains highly controversial. On one hand, TMB has been approved as a companion diagnostic biomarker, and multiple clinical trials have demonstrated its relevance to immunotherapy efficacy (13–15). Multiple studies presented at the 2020 ASCO meeting confirmed the predictive value of TMB in immunization or combination therapy, including KEYNOTE-061 study (16, 17), CONDOR study (18), EAGLE study (19), and EPOC1704 study (20), consolidating TMB as an independent predictor. On the other hand, several investigators have noted that TMB is not a perfect predictor of response to anti-PD-1/PD-L1 therapy, such as in KEYNOTE-158 study (21) and RATIONALE-304 study (22). Clinical studies with RCC (23–25), HPV-positive HNSCC (26), and melanoma receiving anti-PD-1 after recurrence (27) showed that TMB alone neither distinguished responders nor accurately predicted overall survival.

A popular opinion believes that this dispute is mainly caused by the inappropriate thresholds. The quantile-based cutoffs (e.g., median, quartiles) do not accurately reflect the underlying biology of TMB and fail to distinguish patients with their prospective clinical benefits (23, 24, 27, 28). Other conventional categorization methods, which establish a generic TMB threshold based on a single endpoint, reveal only partial therapeutic benefits. A single endpoint cannot fully represent the complexity and efficacy of a disease. Since different single endpoints were used, even on the same cohort of patients, the statistical studies gave inconsistent TMB thresholds, making it difficult for clinicians to make a decision (29). Moreover, the relationship between TMB and ICI benefits may not be uniformly distributed and may also differ across cancer types and corresponding regimens (30–33). Therefore, incorporating multiple efficacy endpoints into multiple categorizations of TMB for various cancers would be more effective in resolving the dispute (34).

Why does the argument still exist when TMB thresholds seem optimal? In data management, we often hear of the “trash in, trash out” principle. Thus, the imprecision of TMB measurements (23, 35, 36) is another crucial or even more dominant fact causing such controversy. Regardless of the various TMB calculation methodologies, none of the mutation callers claim to reach 100% accuracy. They each have their own unique advantages for mutation detection; thus, the errors in TMB measurement cannot be eliminated (37, 38). To avoid the trash-in-trash-out results, it is reasonable to consider TMB errors in threshold optimization, particularly for decision models. Based on the aforementioned multiple-endpoint framework, some study have proposed fault-tolerant statistical models (36) that reduce the instability and bias caused by TMB errors in patient categorization and resulting in improved performance. Although the mutation detection accuracy on each sample may be arbitrarily improved, regardless of the cost, by combining various sequencing technologies, deepening the sequencing depth, etc., it is still hard for the errors to meet the statistical correction assumption of the proposed models. Hence, merely introducing error control or fault tolerance into the decision model is insufficient. The critical error issues from bioinformatics tools that preclude the TMB from being employed in clinical use have not been addressed yet. We are trying to discuss how the issue of errors issue made TMB an ambivalent biomarker, and propose avenues for future research to resolve these tensions.

## 2 Discussion

### 2.1 What are the TMB measurement errors?

Traditionally, when evaluating a bioinformatics tool, researchers use the following performance metrics, including precision, recall, and F1-score, on the average of samples. The goal is to accurately detect mutations of target genes, with a focus on identifying the mutation commonalities among the genome data of patients and maintaining strict control over false positives(FPs), thereby avoiding giving the wrong medicine in targeted therapy. To ensure the detection of gene mutations, bioinformatics has developed numerous variant callers that are sensitive (39) and employs various filters to control FPs. It inevitably results in a large number of false negative(FN) errors while lowering the FP error rate in the final report (40).

In immunotherapy practice, the essence of TMB lies in the total number of mutations rather than a single or multiple targets of interest, regardless of the TMB calculation approaches used. FP and FN errors are equally important in TMB assessment and contribute to the aggregate TMB measurement errors. In TMB errors, the false-positive rate (FPR) is defined as the ratio of the number of FPs to the total number of mutations, whereas the false-negative rate (FNR) is defined as the ratio of the number of FNs to the total number of mutations. FPR and FNR may each obey a non-parametric distribution. They might be a layer-by-layer conditional probability that depends on the types and number of mutations in the sample, the mutation density and composition of the mutations in the particular segments, the design philosophy of the selected caller, the sampling quality, etc. Together, these two complex errors add up to a more complicated and unpredictable TMB measurement error.

### 2.2 What are the effects of complicated TMB errors on the threshold?

Existing TMB thresholds are typically obtained from retrospective investigations of specific immunotherapy patient cohorts. The disadvantage is that the optimized TMB thresholds are frequently less appropriate for broader patient populations, leading to limited generalizability results from sampling bias and measurement inaccuracy within the TMB. Generally, a particular cohort is a small group of patients sampled from a large population based on certain conditional criteria, such as cancer subtypes and enrollment requirements (41), resulting in substantial sampling bias. Due to sampling that violates the principle of randomization, patient cohorts in standard clinical trials are only partially representative of the distribution features of the entire population, resulting in TMB thresholds that are cohort-specific and scalable under extremely demanding conditions. In addition, the risk of measurement error carried by the TMB metric itself influences the transferability of the assigned threshold, even if the sampling population is regularly extended in clinical trials with the expectation that the analytic cohort would precisely characterize the entire distribution. TMB measurement errors can introduce bias in statistical inference (42), which in turn affects decision-making and hinders the effectiveness of therapeutic grouping effects. Here, we use the maximum likelihood estimation (MLE), which is the most popular in statistical inference, as an example to analyze the bias imposed by TMB measurement error on parameter estimation.

The MLE of a parameter θ is generally obtained by solving for the zero solution of a score function (the first-order derivative of the likelihood), i.e., $\Psi \left(\theta \right)=\frac{\partial \ell \left(\theta \right)}{\partial \theta^{T}}=0$ . The basic condition that guarantees the MLE is an unbiased estimator is the expectation unbiasedness of the score function Ψ(·) . Nonetheless, if the TMB observations contain additive error components e , the expectation of the score function must be nonzero since the score function cannot be axisymmetric around the origin.


(1) $$E\left\{\Psi \left(TMB^{*};\Theta \right)\right\}=E\left\{\Psi \left(TMB+e;\Theta \right)\right\}=\int_{-\infty }^{+\infty }\Psi \left(TMB+e;\Theta \right)p\left(e\right)de\neq 0$$

Further, if the error term e is assumed to obey a normal distribution with mean 0 and variance $\Sigma_{e}$ . $Z_{i}$ denotes a vector of covariates, e.g., age, gender, treatment indicator, cancer stage, we take Weibull–Cox PH model as an example, the instantaneous risk for an event depends on $Z_{i}$ and TMB is defined as follow,


(2) $$h\left(t|Z,\;TMB^{*};\theta \right)=\lambda t^{\lambda -1}\exp\left(\beta_{z}^{T}Z+\beta_{m}TMB^{*}\right)$$

Here, the expectation of the score function in Eq (1). can be expressed as


(3) $$\begin{array}{c} E\left\{\text{Δ}Z-T^{\lambda }\cdot \exp\left(\beta_{z}^{T}Z+\beta_{m}TMB^{*}\right)Z\right\}\\ =\text{Δ}Z-T^{\lambda }\cdot E\left\{\exp\left(\beta_{z}^{T}Z+\beta_{m}TMB^{*}+\beta_{m}e\right)Z\right\}\\ =\text{Δ}Z-T^{\lambda }\exp\left(\beta_{z}^{T}Z+\beta_{m}TMB^{*}\right)Z\cdot E\left\{\text{exp}\left(\beta_{m}e\right)\right\}\neq 0 \end{array}$$

where Δ is an event indicator, T denotes the observed event time (such as tumor relapses, progression, death, etc.). The additional term $E\left\{exp\left(\beta_{m}e\right)\right\}$ on the scoring function is caused by the measurement error, leading the naïve estimator to be biased apparently. If the variance fluctuation $\Sigma_{\text{e}}$ can be controlled to approximate zero, the expectation of the score function will converge to zero.


(4) $$\begin{array}{c} E\left\{\text{exp}\left(\beta_{m}e\right)\right\}=\\ \int_{-\infty }^{+\infty }\exp\left(\beta_{m}e\right)\frac{1}{{\text{Σ}}_{e}\sqrt{2\pi }\;}exp\left(-\frac{e^{2}}{2{\text{Σ}}_{e}^{2}\;}\right)de=exp\left(-\frac{{\text{Σ}}_{e}^{2}\beta_{m}^{2}}{2\;}\right)\approx 1 \end{array}$$

(5) $$\begin{array}{c} E\left\{\text{Δ}Z-T^{\lambda }\cdot \exp\left(\beta_{z}^{T}Z+\beta_{m}TMB^{*}\right)Z\right\}\\ \approx E\left\{\text{Δ}Z-T^{\lambda }\cdot \exp\left(\beta_{z}^{T}Z+\beta_{m}TMB\right)Z\right\}\approx 0 \end{array}$$

However, existing approaches barely achieve the variance control. The presence of the unavoidable error term destroys the impartiality nature of the score expectation, resulting in a considerably biased naive MLE estimator, which further affects the downstream TMB threshold determination. The threshold thus obtained is difficult to apply to clinical practice or other historical cohort data.

Furthermore, the mathematical modeling of TMB measurement error is extremely complex. It is related to a number of factors mentioned before, which are interdependent. There exists a complex logical transfer that constitutes nonparametric probability distributions on a layer-by-layer basis. Describing TMB error as a simple Gaussian noise within the conventional decision-making model lacks mathematical rigor, and definitely causes significant confusion in decision-making.

### 2.3 Why is this issue amplified in cancer sequencing data?

Why does this error rate issue seem not to appear in previous sequencing data analysis, especially in a similar genomics problem named population-based data analysis? This is due to the fact that, i) TMB assessment needs to count the total number of detection results, while other application scenarios only need to detect mutations of interest. The switch of needs increases the impact of error rates; ii) the types of mutations in general population are very limited, hence the accuracy of mutation detection software is much higher than that in cancer patients. For example, complex indels only exist in cancer sequencing data. It is a unique form of somatic mutation in tumor samples rarely seen in normal samples; and iii) if the accuracy of a mutation calling tool is sufficiently high, it would be capable of handling the detection and counting tasks very well. For example, the detection accuracy of the 1000 Genomes can easily reach 95% and up. This slight error rate would not affect the counting task since mutations are almost all detected. However, when it comes to complex cancer sequencing data with much lower accuracy, the impact of error rates is further deepened in the counting task. Hence, due to these facts, the original error rate issue has been noticed in cancer sequencing data.

Meanwhile, the clinical need of immunotherapy lies in the ability of variant callers to provide the total mutation count with steady state error rates on a cohort to calculate a fair TMB value for categorization. The variance control of TMB measurement becomes the focus. Despite the factors we discussed in Section 2.1, we focused on the TMB errors from the calling analysis. Bioinformatics software is comparable to a ruler in that it measures the level of patients on a specific dimension related to their immunotherapy prognosis. Just like a ruler, the measurement region should be uniform for all patients. Specifically, the variant callers must have steady performance by maintaining a stable/constant FPR and FNR across patients, as errors are inevitable. In that case, physicians will be able to categorize TMB as a baseline to separate patients into distinct risk groups with varying therapeutic benefits for subsequent clinical decision-making.

**Figure 1:**
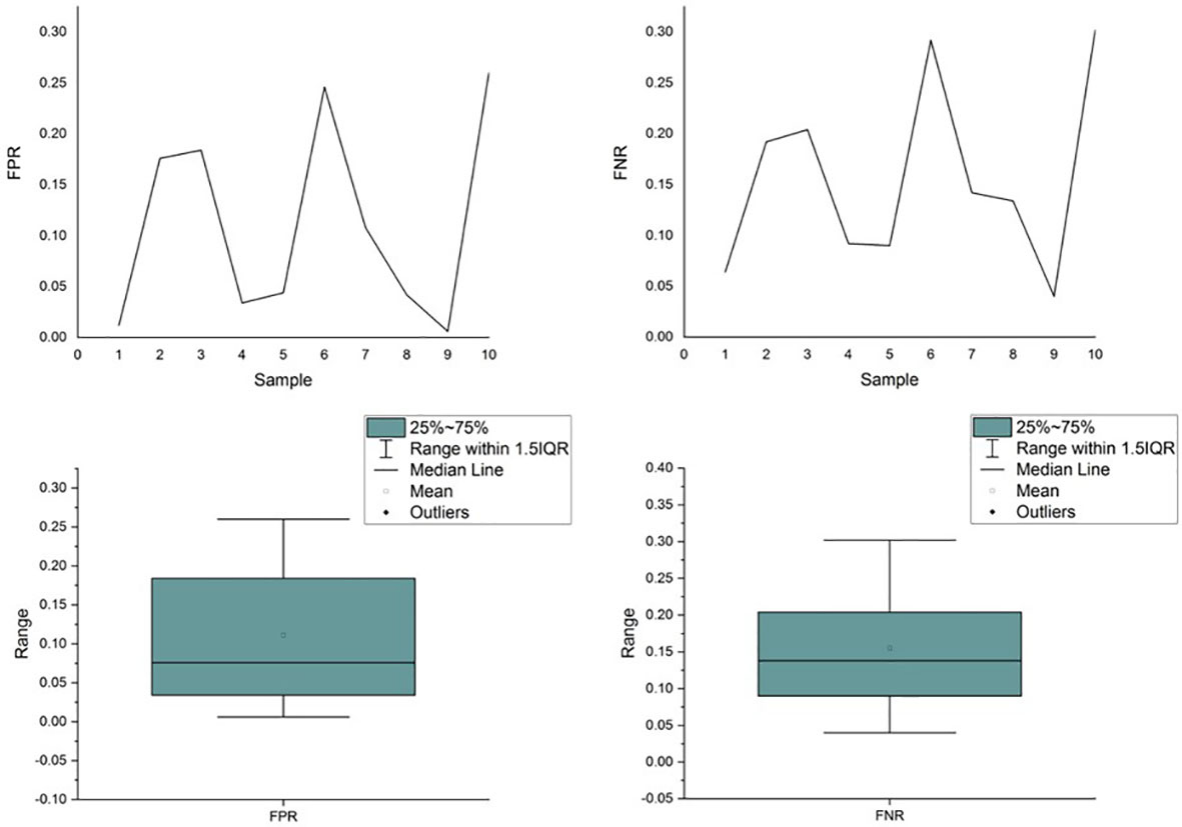
Performance comparison of the caller across simulated samples.

Unfortunately, the existing variant callers are unable to ensure consistent performance across samples, thereby failing to provide a fair TMB for clinical usage. We simulated a data set with 10 samples in which 500 variants, including single-nucleotide variants (SNVs), insertions, and deletions, were randomly planted in a template derived from the reference genome (hg.19). Variant calling was performed using Samtools and Bcftools. As shown in **Figure 1**, the performance of the caller exhibited significant fluctuations in FPR and FNR values across different samples. The coefficient of variation for the FPR and FNR values was 87.90% and 58.61%, respectively, demonstrating that the performance of callers fluctuated significantly as the sample (e.g., the proportion of different variants) varied. Further details of the experiment are presented in **Supplementary 1**.

As compared to the targeted therapy, the differences in the design philosophy make sophisticated bioinformatics tools unable to provide results with steady error rates and minimize TMB errors, thus performing inadequately in immunotherapy guidance. Hence, error control in bioinformatics tools becomes particularly important when using TMB to identify individuals likely to benefit from ICI treatment in a reliable and reproducible manner.

### 2.4 Why does bioinformatics software perform unsteadily across samples?

**Figure 2:**
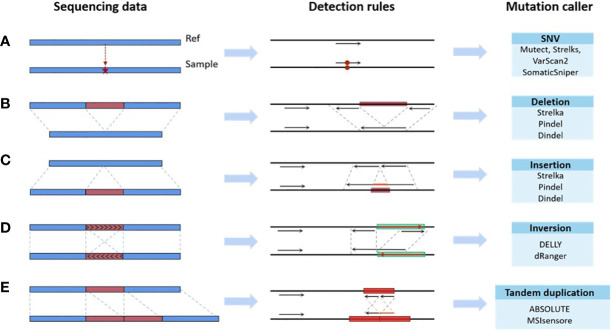
Mapping relationships between features of the sequencing data and mutation types. **(A)** SNV. **(B)** Deletion. **(C)** Insertion. **(D)** Inversion. **(E)** Tandem duplication.

Existing bioinformatics software detects mutations from sequencing data based on rules, which are the mapping relationships between features of the sequencing data (e.g., split reads, abnormal read pairs, sequencing depth, etc.) and outcomes (mutation types), as summarized in **Figure 2**. Taking the deletion variant as an example, in which the sample is missing a fragment relative to the reference genome, there are three types of features when compared to the reference genome: 1) the read depth would be significantly reduced within this deletion region; 2) the insert size of the read pair, which is the spatial distance of the fragment generated by sequencing on both sides of the variant, would be significantly larger; and 3) a read in the sequenced fragment would be split into two fragments with the same alignment direction. Software sets the rules so that a region with these features would be reported as having a deletion variant. These rules are either summarized by researchers *via* experience and observation (43–46) or automatically learned by machine learning algorithms (47–50) based on commonalities among patients. The program reports the detection of a mutation in any genome region whose features fit the predefined rules. Therefore, the accuracy of detection in a certain region relies on the degree of matching between the preset rules of callers and the characteristics of the sequencing data. The mutation types in different samples may not differ greatly, but the proportion of each mutation type may vary significantly. The amount and proportion of mutations whose features do not match preset rules are also different across samples. Using the software with limited predetermined rules to analyze these samples will result in a significant variation in accuracy, as shown in **Figure 1**. The mismatch, caused by the variety of samples and the incompleteness of preset rules, is the fundamental reason for the fluctuation of error rates. It may also help explain why the performance of bioinformatics software differs significantly across populations and even races.

The mutation detection problem for a sample with multiple mutation types may be a non-deterministic polynomial-time hardness problem. That is, when a caller tries to combine all rules to cope with multiple mutation types, it is hard to find a solution to the problem in polynomial-time. As the number of mutation types increases, the number of rules may expand exponentially. Even with the help of machine learning, based on the probabilistic approximately correct (PAC) theory (51), it is only possible to establish an approximately correct set of rules to reduce the generalization error to an acceptable level. Thus, it is not feasible for a variant caller to establish a complete set of recognition rules that encompasses all mappings. Moreover, a general idea of the proof is given below.

**Figure 3:**
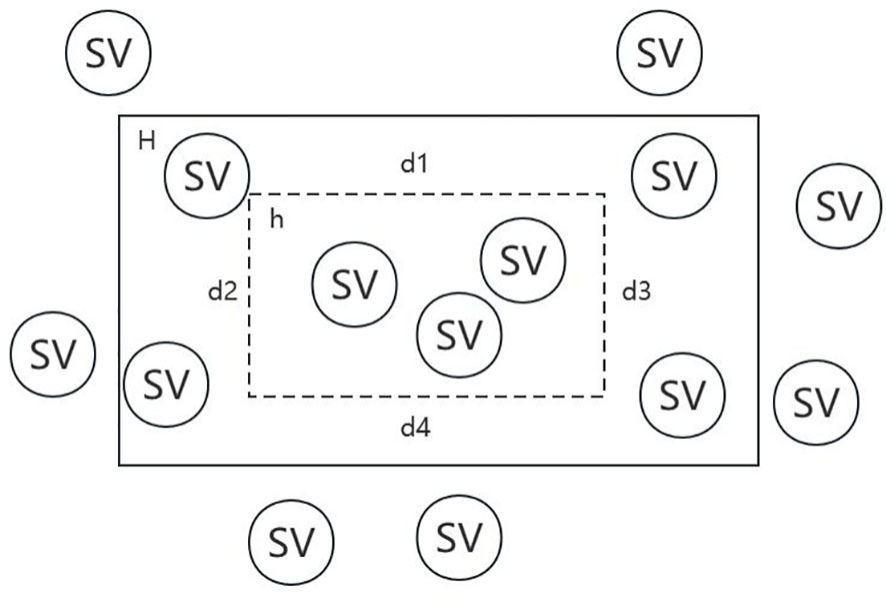
Schematic diagram of target concept and possible hypothesis.

Denote the reals by R, the accuracy control variable by ϵ, the confidence degree control variable by δ, the target concept by H, the possible hypothesis by h and the structural variation by SV. In **Figure 3**, falling within H indicates the reference SV set, while falling within h indicates the call SV set.

For each ϵ∈R , if P[H]≤ϵ , then $\text{R}\left[{\text{C}}_{\text{S}}\right]\leq \text{P}\left[\text{H}\right]\leq \text{ϵ}$ is constant. If P[H]>ϵ , Let’s separate ${\text{d}}_{1},{\text{d}}_{2},{\text{ d}}_{3},{\text{ d}}_{4}\;$ from H, and suppose that their areas are all $\frac{\text{ϵ}}{4}$ , then ${\text{S}}_{{\text{d}}_{1}+{\text{d}}_{2}+{\text{ d}}_{3}+{\text{ d}}_{4}}\leq \text{ϵ}$ . Thus, we can obtain the proposition that if $\text{c}={\text{H}}_{\text{S}}\;$ intersects both ${\text{d}}_{1},{\text{d}}_{2},{\text{ d}}_{3},{\text{ d}}_{4}$ , then H(c)≤ϵ . Its converse proposition is that if H(c)>ε, then ${\text{H}}_{\text{S}}$ does not intersect with at least one of ${\text{d}}_{1},{\text{d}}_{2},{\text{ d}}_{3},{\text{ d}}_{4}$ . Therefore, we can get


(6) $$\begin{array}{l} {\mathbb{P}}_{\text{S}\sim {\text{D}}^{m}}\left[\text{H}({\text{H}}_{\text{S}}\right)>\text{ϵ}]=\cap_{i=1}^{4}\left\{{\text{H}}_{\text{S}}\;\cap {\text{d}}_{\text{i}}=\phi \right\}\\ \leq \cup_{i=1}^{4}\left\{{\text{H}}_{\text{S}}\;\cap {\text{d}}_{\text{i}}=\phi \right\}\\ \leq \sum_{i=1}^{4}{\mathbb{P}}_{\text{S}\sim {\text{D}}^{m}}\left\{{\text{H}}_{\text{S}}\;\cap {\text{d}}_{\text{i}}=\phi \right\}\\ \leq {\left(1-\frac{\text{ϵ}}{4}\right)}^{\text{m}}+{\left(1-\frac{\text{ϵ}}{4}\right)}^{\text{m}}+{\left(1-\frac{\text{ϵ}}{4}\right)}^{\text{m}}+{\left(1-\frac{\text{ϵ}}{4}\right)}^{\text{m}}\\ =4{\text{e}}^{\log{\left(1-\frac{\text{ϵ}}{4}\right)}^{\text{m}}}\\ =4{\text{e}}^{\text{m}\log\left(1-\frac{\text{ϵ}}{4}\right)}\\ \leq 4{\text{e}}^{-\frac{\text{ϵ}}{4}\text{m}}\\ \leq \text{δ} \end{array}$$

In order to ensure that


(7) $${\text{Pd}}_{\text{S}\sim {\text{D}}^{m}}\left[\text{H}\left({\text{C}}_{\text{S}}\right)\leq \text{ϵ}\right]\geq 1-\text{δ}\Leftrightarrow {\text{Pd}}_{\text{S}\sim {\text{D}}^{m}}\left[\text{H}\left({\text{C}}_{\text{S}}\right)>\text{ϵ}\right]\leq \text{δ}$$

Then,


(8) $$4{\text{e}}^{-\frac{\text{ϵ}}{4}\text{m}}\leq \text{δ}\Leftrightarrow \text{m}\geq \frac{4}{\text{ϵ}}\log\frac{4}{\text{δ}}$$

Hence, for each ϵ>0 , δ>0 , if


(9) $$\text{m}\geq \frac{4}{\text{ϵ}}\log\frac{4}{\text{δ}}$$

Then,


(10) $${\text{Pd}}_{\text{S}\sim {\text{D}}^{m}}\left[\text{H}\left({\text{C}}_{\text{S}}\right)\leq \text{ϵ}\right]\geq 1-\text{δ}$$

Thus, this concept class is PAC-learnable. Because of the correlation between some features of the sequencing data (e.g., sequencing depth), an SV can be expressed as an r-term DNF. Applying the result of Pitt and Valiant (52), that r-term DNF are not learnable using r-term DNF as hypotheses in polynomial time unless RP=NP , will complete the proof.

### 2.5 Why the ensemble strategies for bioinformatics software cannot solve this issue?

**Figure 4:**
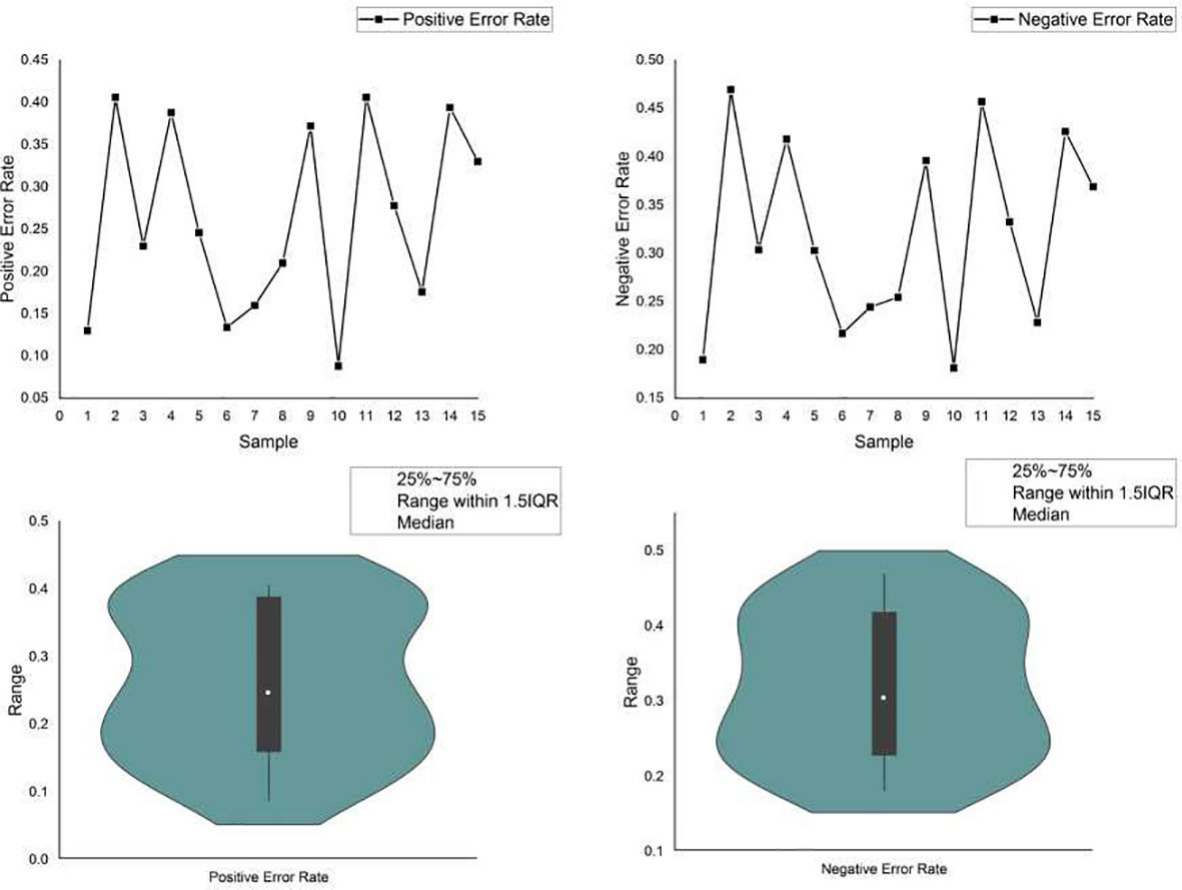
The positive and negative error rate of the ensemble strategy.

Currently, powerful toolkits often adopt ensemble strategies. Multiple mutation detection tools were ensembled, and the consensus voting strategy was used to determine the final detection output. Voting may help to improve the detection of specific candidate targets, hence reducing the risk of FPs. However, it may neglect the important true mutations found by the minority. For example, a delicately designed software detects a mutation that is ignored by all others, yet due to the voting principle, this true mutation is filtered out as a false-positive error, resulting in a false-negative error. We simulated a data set with 15 samples in which 500 variants, including SNVs, insertions and deletions, were randomly planted in a template obtained from the reference genome (hg.19). Three commonly used variant calling flows: samtools + bcftools, freebayes and GATK mutect2 were adopted for the variant calling. We calculated the positive and negative error rates of variant calling using ensemble strategy, as shown in **Figure 4**. Furthermore, we provided two FP and two FN error examples, respectively, caused by the ensemble strategy (**Supplementary 2.3**). The ensemble strategy led to non-negligible errors in both the positive and negative, and the error rate fluctuated significantly, as depicted in **Figure 4**. Through calculation in out experiment, the coefficient of variation of the positive and negative error rates of the ensemble strategy reached 42.79% and 30.86%, respectively, indicating that when the sample changed (e.g., the percentage of various variants changes), the ensemble strategy performance changed accordinssgly (More details are available in **Supplementary 2**). This is because, despite having hundreds of variant callers, their fundamental rules are limited. There are huge overlaps in the basic variant-calling components. In particular, some mutation sites in alleles with low frequency are more likely to be filtered by the voting strategy, hence increasing the risk of FNs. As previously noted, FNs and FPs are equally crucial for TMB. The ensemble strategies voting cannot, therefore, resolve this issue.

## 3 Potential solutions

### 3.1 Software recommendation with improved error variance control performance

Some empirical studies have compared the performance of various variant callers on some benchmarking datasets and demonstrated that most callers have obvious advantages in specific data. These advantages are attributed to the variant caller’s own preset rules, which enable them to handle data with specific mutations. For example, in a benchmark experiment by Kosugi et al. (53), a total of 69 variant callers were tested on second-generation and third-generation sequencing data, both real and simulated. The study revealed that each caller exhibited distinct advantages for specific samples, and no single caller performed optimally across all samples.

As previously noted, based on the clinical need for immunotherapy, bioinformatics mutation detection software is required to take the “personality of samples” into consideration. Technically, the objective is to improve the matching degree between mutations in samples and detection rules. Thus, we can benefit from the idea of the recommender system, which provides suggestions for items that are most pertinent to a particular user. The most suitable bioinformatics software, determined by high matching degree for the samples to be analyzed, can be automatically recommended in a clinical setting. Thus, the recommended bioinformatics software ensures both effectiveness and efficiency in clinical practice, eliminating the need for additional prerequisites. Unlike the traditional recommender system in which the characteristics of users and items are apparent, the bioinformatics software recommender system suggests compatible software for samples whose characteristics are hidsssssden. In this context, we have to mine sample features and evaluate software performance in advance.

Specifically, in the design of traditional recommender systems, researcher guides the process of recommending an item to a user by establishing associations between items and the users. During this process, we can explicitly obtain the user’s gender, age, educational background, and other characteristics to describe a user, as well as the item’s size, color, material, and other attributes to describe an item. Motivated by this, we can achieve software recommendation by finding the characteristics that can describe the sequencing samples and software performance, and subsequently establishing the relationship between them. In this scenario, the user and item are the sample and software, respectively. It appears feasible to implement software recommendations in the same manner as traditional recommender systems. Unfortunately, it is more difficult to obtain the characteristics of a sequencing sample and software than it is to obtain the characteristics of an item or a user. We need to analyze sequencing samples to determine the characteristics that can distinguish among sample differences and software performance variations, such as read length and sequencing depth, among others. Similarly, we need to carry out a large number of experiments to test the performance of the software, in order to achieve a description of the software performance. Therefore, the user and item characteristics in the software recommendation scenario are hidden, which makes this problem challenging.

Some pioneering attempts have been made in this direction. Wang et al. (54) have presented an online SV caller recommendation tool implemented under the meta-learning framework, which automatically recommends the most compatible caller for the input sequencing data. There are some other recommender systems available, such as the SLP-based, ML-KNN-based, and collaborative filtering-based recommendation methods, among others (55). Moreover, the online caller recommendation tool documented in (54) is mainly used for SV caller recommendation. For SNV, indel, CNV, and other scenarios, meta features of the corresponding scenes can also be extracted to guide software recommendation. Therefore, we believe there will be more exciting software recommendation work, such as considering different application scenarios, personalized requirements and so on.

### 3.2 Developing novel bioinformatics tools with error control

Software recommendation is a good way to improve matching degree, however, its performance is still capped by the recommended candidates. Hence, it is still necessary to develop novel bioinformatics tools to achieve control over the error rate. From the perspective of error control, we believe there are several potential studies that warrant further investigation.

1) For some specific types of mutations, such as CNV or MSI, the mapping structure of the rules will not alter even if the variances across samples are substantial. By adjusting the parameters of the rules, it is possible to optimally match the features of different samples with the appropriate rules. Consequently, it is feasible to overcome this kind of problem by incorporating adaptation into the detection scheme. The parameters may self-adjust based on the features of the samples, thereby increasing the degree of matching in an adaptive manner. Some pioneer researches have made attempts. Taking CNV detection as an example, the size of sliding window is the crucial factor impacting the detection precision. Xuwen et al. (56) have presented a CNV caller with a dynamic sliding window that automatically adjust the window size based on the length of CNV to achieve the optimal setting. The adaptive window with self-adopted size makes it capable of handling CNVs with various lengths ranging from kb-scale to chromosome-arm level.

2) From the perspective of the control system, this problem can be viewed as a detection quality control problem. By setting the detection error as the adaptive control goal, it is feasible to introduce the adaptive control mechanism into the algorithm design to utilize the error feedback information. Hence, the detection algorithm would have the adaptability of dynamic adjustment and automatic matching of sequencing sample characteristics. However, there are two challenges that need to be tackled here. First of all, how do we establish the model for this detection quality control problem? Establishing accurate models of systems with nonlinear characteristics has been one of the most challenging and critical challenges in control science. Meanwhile, the mutation detection process is a special process of statistical analysis of static sequencing data and outputting results. The traditional modeling methods of physical systems cannot be directly applied in this context. Instead, it is necessary to study the key factors affecting quality control, such as the multi-dimensional sample characteristics and detection rule parameters and define the dynamic characteristics of the detection process. Secondly, how do we design a control strategy with adaptiveness and robustness to achieve the quality control of mutation detection? A complex nonlinear system with high uncertainties is one of the classical control problems in the field of nonlinear systems. There are numerous control approaches that have been studied and developed. However, the field currently lacks mathematical tools to uniformly deal with nonlinear systems, and a general optimal design solution is yet to be established, which needs to be analyzed and handled case-by-case. It needs to study how to introduce adaptive mechanisms into mutation detection methods, design control strategies based on the error feedback information and system model, and guide the design of adaptive detection algorithms.

3) Another one is to deal with multiple types of mutations simultaneously, each with its own set of rules. By adopting the concept of the recommender system in the selection of bioinformatics software, a sample may be viewed as a collection of different mutations with varying proportions and types. The sequencing data is divided into a series of intervals, with each interval containing just one mutation. These intervals are then clustered. The best combination of bioinformatics software is selected by recommending the optimally matched rule sets for the clustered classes.

These studies may be further ensembled to address more complex cases, such as the overlap of different mutations within a single interval window. As an added value, this might shed light on why bioinformatics tools have inconsistent performance when detecting mutations in data from individuals of different races.

### 3.3 New threshold optimization methods to better consider the error

The threshold optimization method considering FN and FP errors is also a viable research topic for addressing the TMB issue. From the perspective of the errors, its fundamental concept is to correct the number of mutations detected within any specific interval of the samples in the statistical framework.

Thereby, the machine learning algorithm may be employed to learn the features of TMB that cannot be directly observed during detection, to predict the deviation risk of detected mutations, and to thereafter monitor the risk outliers among samples. Based on the predicted risk, the new threshold optimization methods would be able to assist in immune decision-making modeling, which will facilitate clinically precise diagnosis and therapy.

Compared to the existing multiple-end statistical models, which directly estimate the TMB error of each sample, this model uses machine learning to predict FPs and FNs instead, tracing the errors back one step farther. The information loss is reduced. Thus, the errors are more effectively handled across samples.

## 4 Conclusion

This article investigates the underlying reasons why TMB becomes an ambivalent biomarker. The definition of TMB error is given first. Then the requirements of immunotherapy for bioinformatics tools are analyzed to trace the source of the TMB error. The simulation results also demonstrate that the variant caller performs unsteadily across samples and cannot fulfill the requirements. The effects of TMB error on threshold and the reasons why error issue is amplified in cancer sequencing data are also discussed. By analyzing the design philosophy behind callers, the conflict between the incompleteness of biostatistics rules and the variety of clinical samples is the critical issue that renders TMB an ambivalent biomarker. Additionally, the article also proposes potential research topics in order to address the conflict issue.

## Data availability statement

Publicly available datasets were analyzed in this study. This data can be found here: https://www.internationalgenome.org.

## Author contributions

JW and YQL contributed to conception and design of the study; SW performed the experiments and analyzed the data; XYZ, XL, XPZ, XQL and XX helped perform the analysis with constructive discussions; YQL and JW wrote the first draft of the manuscript; YW, SW and YFL wrote some sections of the manuscript. All authors contributed to the article and approved the submitted version.
