# Supplementary material for: What makes TMB an ambivalent biomarker for immunotherapy? A subtle mismatch between the sample-based design of variant callers and real clinical cohort
Source: Front Immunol. 2023 May 25;14:1151224. doi: 10.3389/fimmu.2023.1151224 (PMC10248171; doi:10.3389/fimmu.2023.1151224)
Supplement: Supplementary file 1 [file DataSheet_1.docx]

Supplementary Material

What makes TMB an ambivalent biomarker for immunotherapy? A subtle mismatch between the sample-based design of variant callers and real clinical cohort

Yuqian Liu^1,2,†^, Shenjie Wang^1,2,†^, Yixuan Wang^2,3,†^, Yifei Li^1,^, Xiaoyan Zhu^1,2^, Xin Lai^1,2^, Xuanping Zhang^1,2^, Xuqi Li^4,^*, Xiao Xiao^2,5^, Jiayin Wang^1,2,^*

*** Correspondence:** Jiayin Wang: wangjiayin@mail.xjtu.edu.cn

Xuqi Li: lixuqi@163.com

# Caller Stability Experiments

## Data Preparation

We chose hg19.fa as the reference genome to generate the simulated dataset. A 10Mbps region was randomly sampled from the chromosome1 first. And 500 variants were randomly planted in this region, including single-nucleotide variants (snvs), insertions and deletions. We repeated the experiment ten times, randomly adjusting the percentages of snvs, insertions and deletions for each experiment, and obtained ten different simulated samples. The simulation tool used in our experiments is GSDcreator. For the variants simulation, GSDcreator replaces bases at the specified positions on the template sequence according to the corresponding mutation rates, and then outputs the template sequence with variants planted on. The align tool used in the experiment is BWA.

## Calling Pipeline

For the variants calling, we adopted Samtools and Bcftools. First, we use the mpileup function of Samtools to generate the pileup file. Then, we generate vcf file based on the pileup file, using the call function of Bcftools. The pipeline is shown in the Supplemental Figure 1.

## Discussion

Based on the results, we can find that the caller performance has large fluctuations in FPR and FNR values on different samples. Furthermore, we calculated the coefficient of variation of FPR and FNR, respectively. The coefficient of variation is a normalized measure of the degree of dispersion of the probability distribution, defined as the ratio of standard deviation to the mean, which can reflect the degree of dispersion of the data. Through calculation, the coefficient of variation of FPR and FNR values of the variants caller in our experiment reached 87.90% and 58.61%, respectively, indicating that when the sample changes (e.g., the percentage of various variants changes), the caller performance changes accordingly.


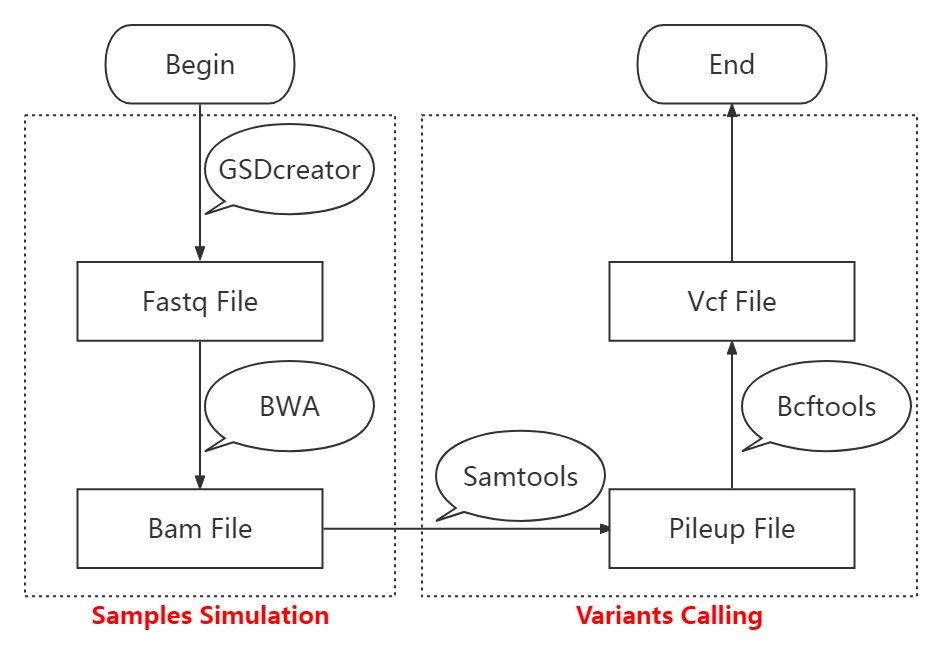


**Supplementary Figure 1.** Diagram of calling pipeline.

# Ensemble Strategy Experiments

## Data Preparation

Again, we chose hg19.fa as the reference genome to generate the simulated dataset. A 10Mbps region was randomly sampled from the chromosome1 first. And 500 variants were randomly planted in this region, including single-nucleotide variants (snvs), insertions and deletions. We repeated the experiment fifteen times, randomly adjusting the percentages of snvs, insertions and deletions for each experiment, and obtained fifteen different simulated samples. The simulation tool used in our experiments is GSDcreator. For the variants simulation, GSDcreator replaces bases at the specified positions on the template sequence according to the corresponding mutation rates, and then outputs the template sequence with variants planted on. The align tool used in the experiment is BWA.

## Calling Pipeline

For the variants calling, we adopted three commonly used variants calling flows: samtools + bcftools, freebayes and GATK mutect2. First, we run three calling flows separately on each sample in turn and get the vcf file for each caller. Then, we screened the variants detected by at least two or more calling flows at the same time and combined them to obtain the ensemble variants calling results. The pipeline is shown in the Supplemental Figure 2.


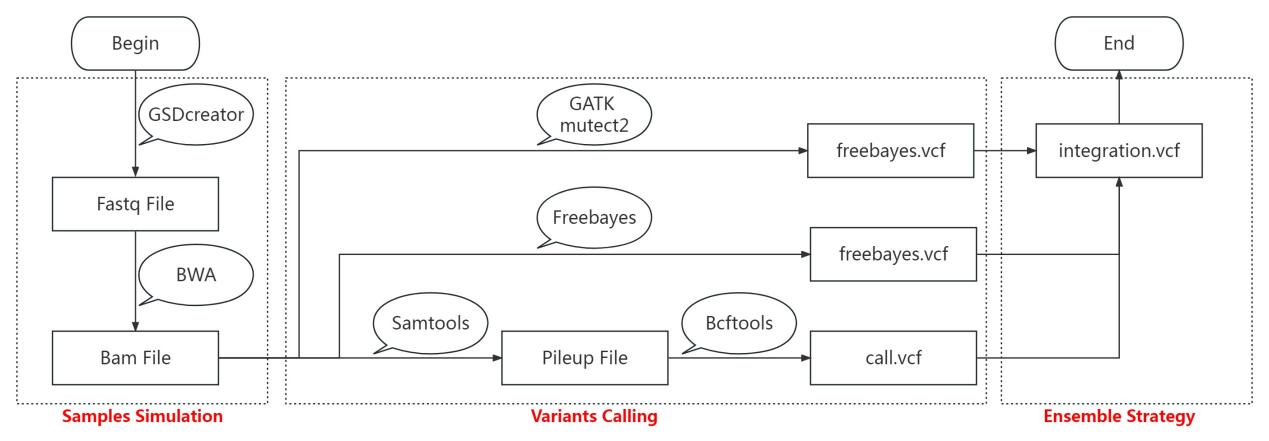


**Supplementary Figure 2.** Diagram of ensemble strategy calling pipeline.

## Examples of Ensemble Strategy Failure

We give two false positive and two false negative examples respectively, as shown below.

(1)False Positive：The ensemble strategy determines positive, but it is actually negative.

False Positive 1：SNV variant (C>T), calling Flow1 and calling Flow2 determine positive, calling Flow3 determines negative.

calling Flow1: samtools + bcftools


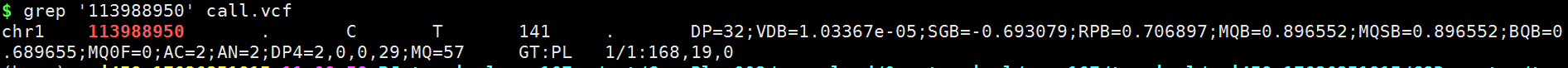


calling Flow2: freebayes


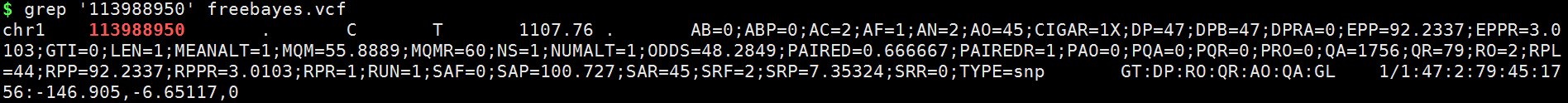


calling Flow3: GATK mutect2


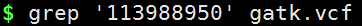


False Positive 2：SNV variant (C>T), calling Flow1 and calling Flow3 determine positive, calling Flow2 determines negative.

calling Flow1: samtools + bcftools


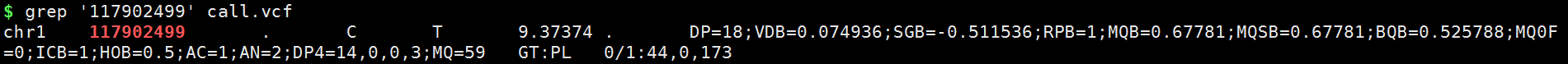


calling Flow2: freebayes


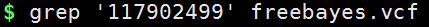


calling Flow3: GATK mutect2


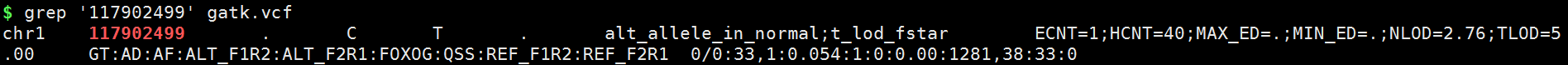


(2)False Negative：The ensemble strategy determines negative, but it is actually positive.

False Negative 1：INDEL variant (AA>AGGACCTA), calling Flow1 and calling Flow3 determine negative, calling Flow2 determines positive.

calling Flow1: samtools + bcftools


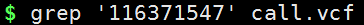


calling Flow2: freebayes


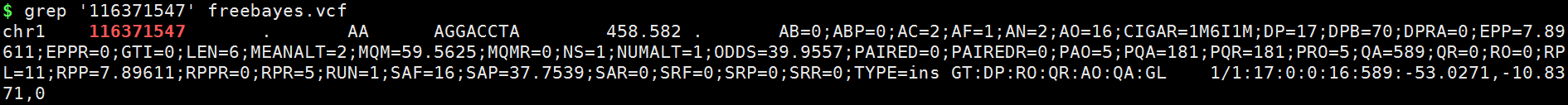


calling Flow3: GATK mutect2


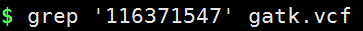


False Negative 2：INDEL variant (t>tTGCATTAAAC), calling Flow2 and calling Flow3 determine negative, calling Flow1 determines positive.

calling Flow1: samtools + bcftools


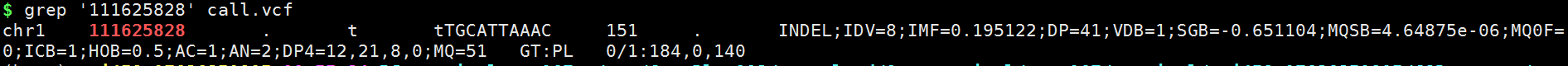


calling Flow2: freebayes


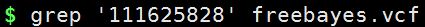


calling Flow3: GATK mutect2


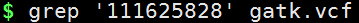


## Discussion

Based on the results, we can find that there are non-negligible errors in both positive and negative with ensemble strategy, and the error rate fluctuates greatly. Through calculation, the coefficient of variation of positive and negative error rate of the ensemble strategy in our experiment reached 42.79% and 30.86%, respectively, indicating that when the sample changes (e.g., the percentage of various variants changes), the ensemble strategy performance changes accordingly. This is because, despite having hundreds of variant callers, their fundamental rules are limited. There are huge overlaps in the basic variant-calling components. In particular, some mutation sites in alleles with low frequency are more likely to be filtered by the voting strategy, hence increasing the risk of false negatives.
